# The Silent Sentinel’s Breach: Middle Lobe Syndrome Caused by Endobronchial Rupture of Mediastinal Tuberculous Lymphadenitis

**DOI:** 10.7759/cureus.115134

**Published:** 2026-08-25

**Authors:** Houda Snene, Monia Attia, Imen Alioua, Nadia Mehiri, Bechir Louzir

**Affiliations:** 1 Department of Pulmonology and Allergology, Mongi Slim University Hospital, Tunis El Manar University, La Marsa, Tunis, TUN; 2 Department of Medical Imaging, Mongi Slim University Hospital, Tunis El Manar University, La Marsa, Tunis, TUN

**Keywords:** bronchodular fistula, endobronchial tuberculosis, mediastinal lymphadenitis, middle lobe syndrome, tuberculosis

## Abstract

Middle lobe syndrome (MLS) is characterized by recurrent or chronic collapse of the right middle lobe of the lung. While non-vascular inflammatory causes dominate, endobronchial rupture of mediastinal tuberculous lymphadenitis causing obstructive MLS is an under-recognized entity that presents significant diagnostic challenges. In this context, we report the case of a 24-year-old male immigrant with no past medical history who presented with cough and right-sided chest pain. Chest radiography revealed right middle lobe alveolar opacity. He received empirical levofloxacin without radiological follow-up. Four months later, he experienced symptom recurrence associated with weight loss, asthenia, and night sweats. Repeat chest radiography revealed persistent middle lobe consolidation with ipsilateral pleural effusion. Sputum smear for acid-fast bacilli and PCR for *Mycobacterium tuberculosis* were negative. Chest CT confirmed necrotic mediastinal lymph nodes with endobronchial rupture into the middle lobe bronchus and cavitated middle lobe consolidation. Flexible bronchoscopy demonstrated mucosal infiltration and pearly-white stenosis of the middle lobar bronchus. Endobronchial biopsies revealed necrotizing epithelioid and giant-cell granulomatous inflammation. The diagnosis of obstructive MLS secondary to endobronchial tuberculosis following lymph node rupture was established, and antituberculosis therapy was initiated. In conclusion, tuberculous lymphadenitis with broncho-nodular fistulization should be considered in the etiology of obstructive MLS. Initial empirical fluoroquinolone treatment can mask bacteriological detection, highlighting the essential diagnostic role of endobronchial biopsies.

## Introduction

Middle lobe syndrome (MLS) refers to a spectrum of conditions that lead to chronic or recurrent atelectasis or consolidation of the right middle lobe [1, 2]. Mechanistically, MLS is categorized into obstructive and non-obstructive types [1]. The unique anatomical features of the middle lobe bronchus, which are its small diameter, acute take-off angle, and surrounding collar of lymphatic channels, render it particularly vulnerable to extrinsic compression and secondary endobronchial involvement [1, 2].

Although inflammatory conditions (47%) and malignant tumors (22%) represent the primary etiologies of MLS, tuberculous origin accounts for approximately 9% of cases [1, 3]. Mediastinal tuberculous lymphadenitis eroding directly into the tracheobronchial tree represents a rare and challenging cause of obstructive MLS [3-5]. Here, we report a case of obstructive MLS resulting from endobronchial rupture of necrotic hilar/mediastinal tuberculous lymph nodes and provide a brief literature review focused on clinical management and diagnostic caveats.

## Case presentation

**Figure 1:**
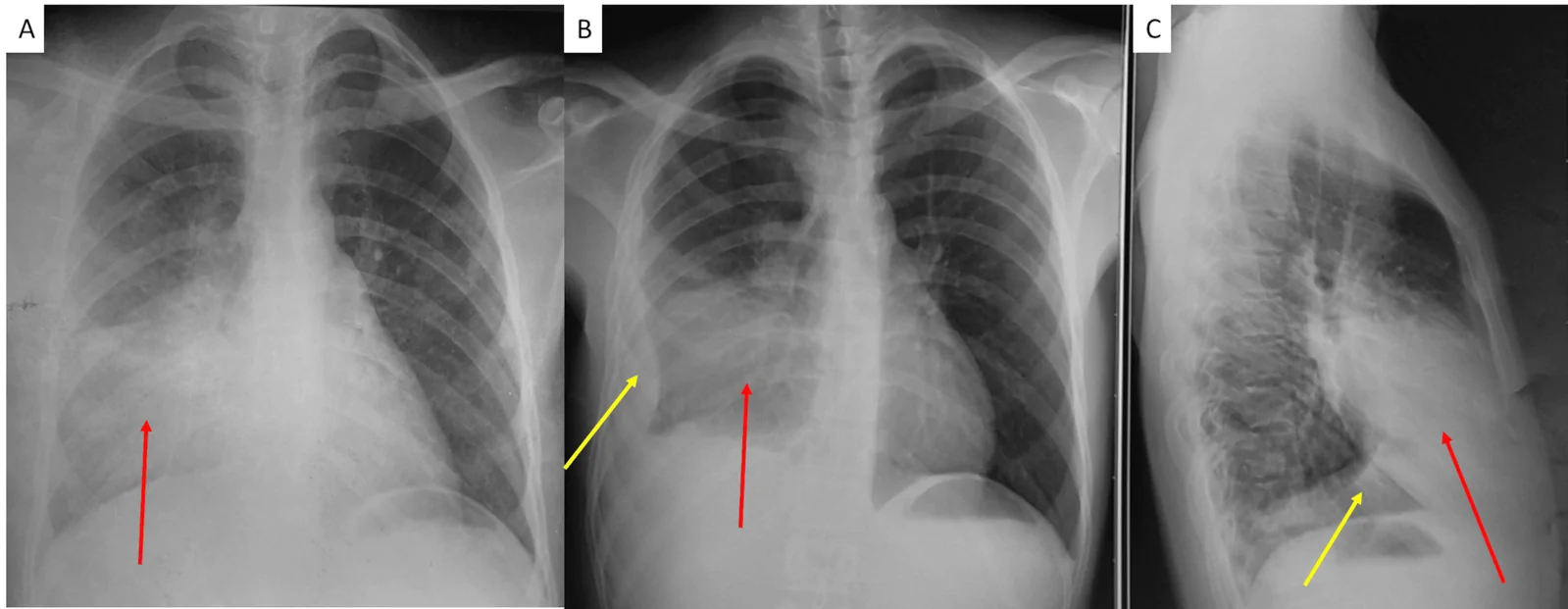
(A) Initial chest radiography showing alveolar opacity in the right middle lobe (red arrow). (B and C) Repeat chest radiography (B: frontal view; C: lateral view) four months later demonstrating persistent right middle lobe consolidation (red arrow) with ipsilateral pleural effusion (yellow arrow).

A 24-year-old male immigrant living in a precarious social situation with no previous medical history presented to a community healthcare physician with a persistent cough and right-sided chest pain. Initial chest radiography revealed alveolar opacity localized in the right middle lobe (Figure 1A). He was treated empirically with a standard course of levofloxacin without subsequent post-treatment radiological evaluation. Four months after the completion of the initial antibiotic course, the patient experienced a clinical relapse, characterized by recurrent cough, right chest pain, profuse night sweats, and significant constitutional symptoms, including asthenia, anorexia, and weight loss. Physical examination revealed a hemodynamically stable patient without respiratory distress. Laboratory investigations are detailed in Table 1. The analyses revealed a normal white blood cell count without lymphopenia, normocytic normochromic anemia (hemoglobin: 11.2 g/dL), significantly elevated C-reactive protein (153.33 mg/L), and overall preserved renal and liver functions.

**Table 1:** Initial laboratory investigations on admission

Laboratory parameter	Patient's result	Reference range	Units
Hematology (Complete blood count)			
White blood cells	6.65	4.00 – 10.00	x 10^9^/L
Neutrophils (Absolute)	3.98	1.70 – 7.10	x 10^9^/L
Lymphocytes (Absolute)	2.16	1.50 – 4.10	x 10^9^/L
Hemoglobin	11.2	12.0 – 18.0	g/dL
Mean corpuscular volume	74.9	80.0 – 100.0	fL
Mean corpuscular hemoglobin	24.4	26.0 – 34.0	pg
Mean corpuscular hemoglobin concentration	32.6	32.0 – 36.0	g/dL
Platelets	402	150 – 450	x 10^9^/L
Inflammatory marker			
C-Reactive Protein	153.33	< 7	mg/L
Renal function and electrolytes			
Urea	1.66	2.50 – 7.50	mmol/L
Creatinine	70.54	53.00 – 115.00	µmol/L
Sodium	134	136 – 145	mmol/L
Potassium	4.36	3.50 – 4.90	mmol/L
Chloride	99	98 – 107	mmol/L
Uric Acid	264.46	200.00 – 420.00	µmol/L
Hepatic function and enzymes			
Total bilirubin	5.1	5 – 17	µmol/L
Direct bilirubin	1.0	< 5	µmol/L
Aspartate aminotransferase	37	5 – 40	IU/L
Alanine aminotransferase	28	5 – 40	IU/L
Alkaline phosphatase	106	40 – 129	IU/L
Gamma-glutamyl transferase	83	5 – 30	IU/L

A second chest X-ray revealed persistent consolidation in the right middle lobe, associated with a small ipsilateral pleural effusion (Figure 1B, 1C).

**Figure 2:**
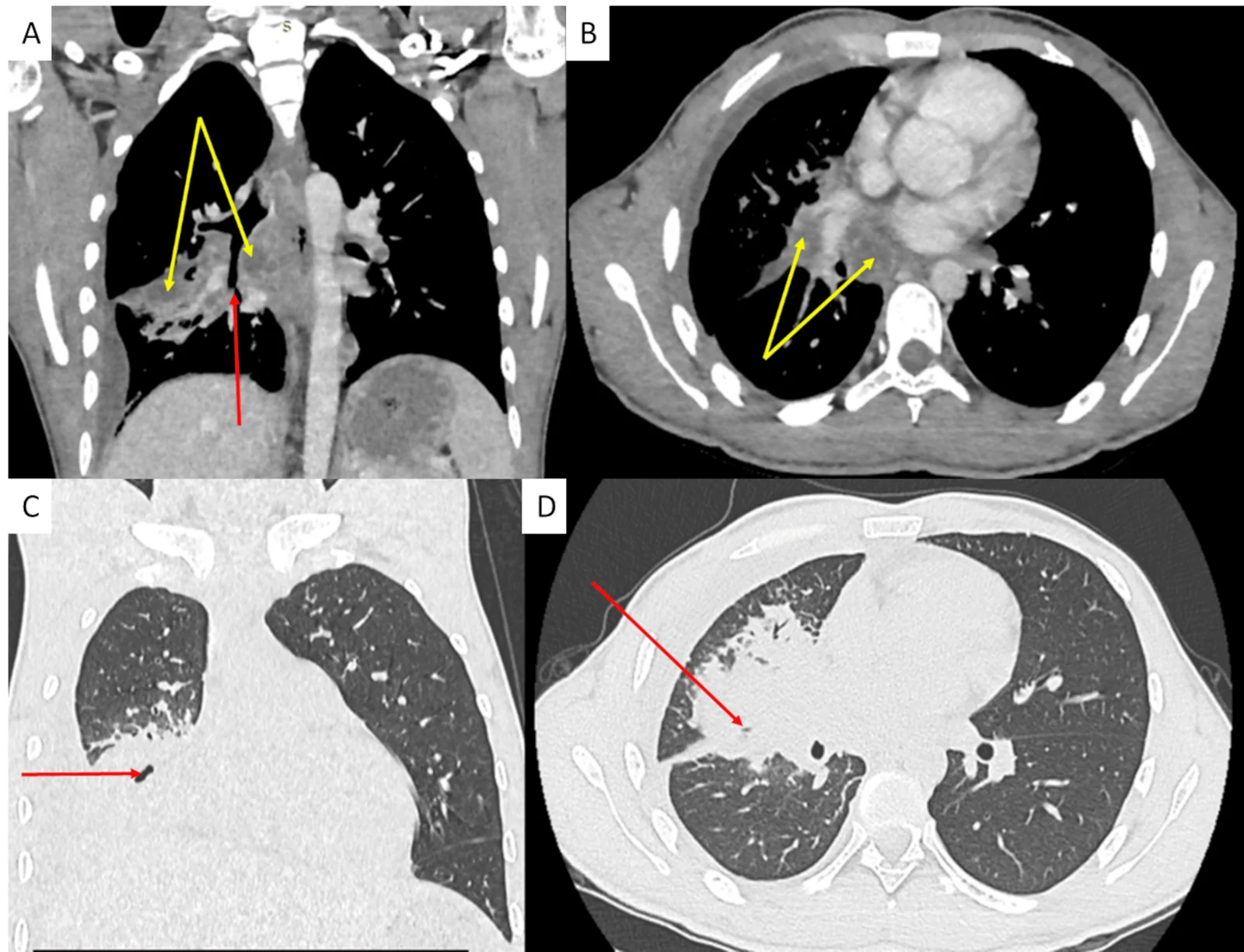
Mediastinal window (A: coronal view; B: axial view) showing necrotic hilar/subcarinal lymph nodes (yellow arrows) with endobronchial rupture into the middle lobe bronchus (red arrow). Parenchymal window (C: coronal view; D: axial view) showing cavitated consolidation in the right middle lobe (red arrow).

Sputum smear for acid-fast bacilli (AFB) and PCR for Mycobacterium tuberculosis were negative. Pleural fluid aspiration could not be performed due to minimal fluid volume on chest ultrasound, and the tuberculin skin test (TST) was non-reactive. Contrast-enhanced chest computed tomography (CT) in the mediastinal window revealed necrotic hilar and subcarinal lymph nodes with direct endobronchial rupture into the lumen of the middle lobar bronchus (Figure 2A, 2B). Parenchymal window settings showed dense consolidation of the right middle lobe, containing internal cavitations (Figure 2C, 2D).

**Figure 3:**
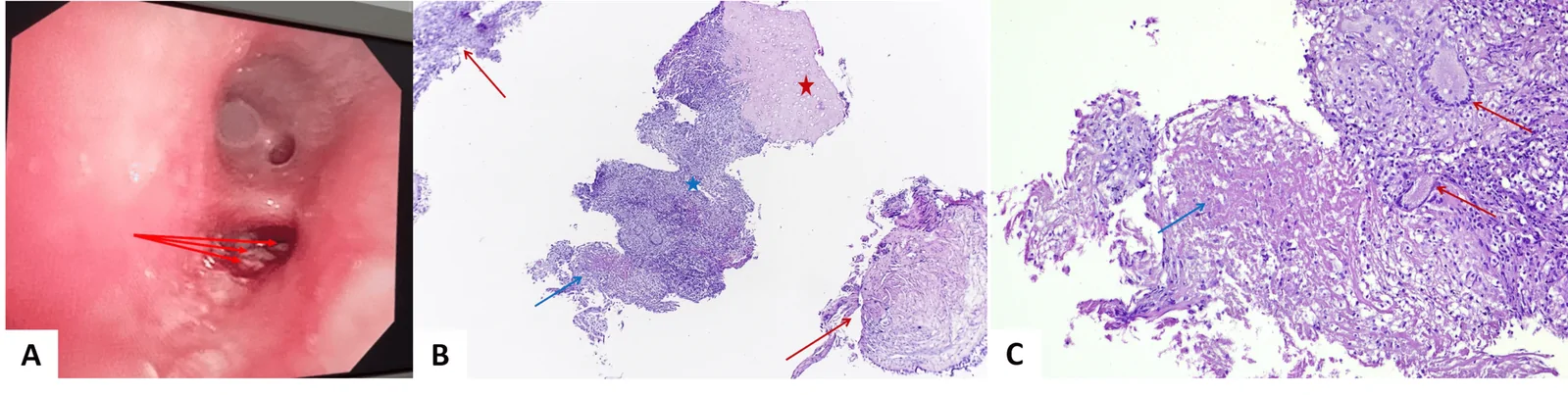
(A) Flexible bronchoscopy showing narrowing and pearly white mucosal infiltration of the middle lobar bronchus (red arrows). (B) Microscopic examination: Three biopsy samples, two concerned altered bronchial mucosa (red arrow). The middle one includes cartilage (red star) and contains granulomas (blue star) and eosinophilic necrosis on its lower end (blue arrow) (Hematoxylin-eosin X100). (C) Microscopic examination: Necrotizing granulomatous inflammation featuring epithelioid histiocytes, Langhans-type giant cells (red arrows), and a surrounding lymphocytic infiltrate, adjacent to a zone of eosinophilic caseous necrosis (blue arrow) (Hematoxylin-eosin X250).

Flexible bronchoscopy demonstrated marked narrowing of the right middle lobar bronchus due to transmucosal infiltration with focal pearly-white mucosal changes (Figure 3A). The remaining bronchial tree exhibited normal mucosa. Endobronchial biopsies were performed at the site of stenosis. AFB testing on bronchial aspirate fluid returned negative. However, histopathological examination of the endobronchial biopsies demonstrated ulcerated mucosa with dense inflammatory infiltrates and multiple epithelioid giant-cell granulomas exhibiting focal caseous necrosis, with no evidence of malignancy (Figure 3B, 3C).

A final diagnosis of obstructive middle lobe syndrome secondary to endobronchial tuberculosis following mediastinal lymph node rupture was established. Standard antituberculosis therapy (rifampicin, isoniazid, pyrazinamide, and ethambutol) was initiated, leading to progressive clinical improvement.

## Discussion

While MLS is a well-described clinical entity (accounting for 2-5% of all adult pulmonary referrals) [1], endobronchial erosion of mediastinal tuberculous lymphadenitis remains a distinctly rare mechanism in adults compared to pediatric populations [2, 3]. In pediatric series, enlarged caseous lymph nodes easily erode the compliant pediatric bronchial wall. Conversely, adult tracheobronchial erosion requires chronic transmural inflammatory destruction of mature cartilaginous rings, ultimately leading to luminal compromise and bronchial stenosis [2, 4, 5]. Recent cohort studies evaluating predictors of endobronchial tuberculosis emphasize that acute mucosal involvement can rapidly progress to irreversible endobronchial scarring if the diagnosis is delayed [3]. The anatomical susceptibility of the right middle lobe bronchus is attributed to three interconnected anatomical factors [1, 6]. Firstly, the bronchus is characterized by a relatively long caliber and a narrow lumen, measuring only 1.5 to 2 cm, which makes it inherently susceptible to rapid extrinsic compression and luminal occlusion [1]. Secondly, its acute take-off angle from the bronchus intermedius significantly limits effective collateral ventilation from adjacent pulmonary segments [6]. Lastly, the airway is surrounded by a dense peribronchial lymphatic chain that receives drainage from both the middle and right lower lobes, thereby subjecting it to direct extrinsic inflammatory pressure during regional lymphadenopathy [1]. In tuberculous lymphadenitis, nodal expansion causes initial extrinsic mechanical compression (stage 1). As central liquefactive caseous necrosis progresses, periadenitis induces transmural erosion into the contiguous bronchial wall, forming a broncho-nodular fistula (Stage 2) [2, 4, 7]. Contrast-enhanced chest CT plays a crucial diagnostic role by depicting central airway involvement, tree-in-bud opacities, and characteristic peripheral rim enhancement of necrotic mediastinal lymph nodes [8, 9]. In a landmark imaging series of central airway tuberculosis, Moon et al. [8] emphasized the key CT features of bronchial wall thickening, luminal narrowing, and adjacent mediastinal adenopathy. Furthermore, in a dedicated radiological series of adult patients with tuberculous bronchonodal fistulas, Park et al. [9] demonstrated that contrast-enhanced CT is crucial for detecting nodal necrosis and fistulous tract formation between mediastinal lymph nodes and adjacent bronchial lumens, which frequently causes secondary endobronchial involvement and airway occlusion. The discharge of necrotic caseum into the lumen creates secondary endobronchial tuberculosis and intrinsic airway occlusion [3, 5, 7]. To put our patient's presentation into perspective, Table 2 provides a comparative summary alongside both an individual case report in a young adult [10] and a retrospective series of 22 adult patients with middle lobe syndrome secondary to endobronchial tuberculosis [11]. While Kim et al. demonstrated that edematous-type mucosal involvement in elderly patients accounts for the majority of adult presentations, our case, together with Garg et al., highlights the distinct clinical scenario of transmural broncho-nodular erosion in young males. Crucially, across all cohorts, non-invasive AFB smear sensitivity remains notoriously low, reaffirming the indispensable diagnostic yield of bronchoscopic tissue sampling [3, 7, 10, 11].

**Table 2:** Comparative summary of tuberculous middle lobe syndrome: Current case vs. isolated report in a young adult (Garg et al.) and a retrospective adult cohort (Kim et al.)

Clinical / Diagnostic parameter	Current case (2026)	Garg et al. (2015) [10]	Kim et al. series (2008) [11]
Study type / Population	Case report (n=1) / 24-year-old male	Case report (n=1) / 19-year-old male	Retrospective cohort (n=22) / Female predominance (18 F / 4 M), mean age: 70 years
Initial symptoms	Cough, pleuritic chest pain, deterioration of general condition; prior fluoroquinolone exposure	Cough, fever, right-sided chest pain, weight loss	Cough and sputum (most common); dyspnea; 2 patients were asymptomatic
CT imaging findings	Cavitated RML consolidation, necrotic subcarinal lymph nodes	RML collapse/consolidation, necrotic mediastinal lymphadenopathy	RML consolidation / atelectasis / patchy opacities
Bronchoscopic appearance	Pearly mucosal infiltration, luminal occlusion of the RML bronchus	Narrowed RML bronchus, swollen hyperemic mucosa, cheesy secretions	Edematous-hyperemic type (15/22), active caseous type (6/22), tumorous type (1/22)
Microbiological yield (AFB)	Sputum and bronchial fluid: Negative	Sputum smear: Negative; GeneXpert PCR: Positive	Negative in 77% of cases (positive in only 1/15 in the edematous subtype)
Histopathological findings	Endobronchial biopsy: Caseating granulomatous inflammation	Endobronchial biopsy: Caseating granulomatous inflammation	Bronchial biopsy: Epithelioid / caseating granuloma (diagnostic in 16/22)
Treatment and outcome	2HRZE / 4HR regimen; complete clinical and radiological resolution	Standard anti-TB regimen; full clinical and radiological resolution	Complete or partial radiological improvement in the majority of patients

As highlighted in Table 2, a critical diagnostic finding illustrated by our case and mirrored in the literature is the diagnostic delay frequently observed in endobronchial tuberculosis due to its atypical presentation [12], which is often further compounded by empiric fluoroquinolone monotherapy [13]. Fluoroquinolones (such as levofloxacin or moxifloxacin) possess potent activity against M. tuberculosis [13]. Empirical administration for suspected community-acquired pneumonia transiently reduces the bacterial burden in respiratory secretions, leading to false-negative AFB smears and PCRs [12, 13]. However, partial therapy is insufficient to eradicate deep-seated nodal tissue infection, promoting clinical relapse weeks to months later [13]. In such scenarios, histopathological identification of necrotizing epithelioid granulomas via endobronchial biopsy remains the definitive diagnostic gold standard [3, 7, 12].

## Conclusions

This clinical observation and literature review emphasize that broncho-nodular fistulisation of necrotic mediastinal tuberculous lymphadenitis must remain a key differential diagnosis in patients presenting with obstructive middle lobe syndrome, especially in young adults from endemic regions. Fluoroquinolones should be used with extreme caution in patients with unconfirmed respiratory infections. When microbiological assays are negative, flexible bronchoscopy remains essential in this setting to rule out an underlying tumor, establish the diagnosis, and prevent unnecessary surgical intervention.
